# Rooting and acclimatization of micropropagated marubakaido apple rootstock using *Adesmia latifolia* rhizobia

**DOI:** 10.1186/2193-1801-2-437

**Published:** 2013-09-04

**Authors:** Aleksander Westphal Muniz, Enilson Luiz de Sá, Gilberto Luíz Dalagnol, João Américo Filho

**Affiliations:** Embrapa, Rodovia AM 10 Km 29, CEP 69010-970 Manaus- AM, Brazil; Departamento de Microbiologia do Solo, Universidade Federal do Rio Grande do Sul (UFRGS), Avenida Bento Gonçalves 7712, CEP 91540-000 Porto Alegre-RS, Brazil; Epagri, Rua José Godinho SN, CEP 88502-970 Lages-SC, Brazil

**Keywords:** Auxins, Micropropagation, Apple, Indoleacetic acid

## Abstract

In vitro rooting and the acclimatization of micropropagated rootstocks of apple trees is essential for plant development in the field. The aim of this work was to assess the use of rhizobia of *Adesmia latifolia* to promote rooting and acclimatization in micropropagated Marubakaido apple rootstock. An experiment involving *in vitro* rooting and acclimatization was performed with four strains of rhizobium and two controls, one with and the other without the addition of synthetic indoleacetic acid. The inoculated treatments involved the use of sterile inoculum and inoculum containing live rhizobia. The most significant effects on the rooting rate, primary-root length, number of roots, root length, fresh-shoot biomass, and fresh-root biomass were obtained by inoculation with strain EEL16010B and with synthetic indole acetic acid. However, there was no difference in the growth of apple explants in the acclimatization experiments. Strain EEL16010B can be used to induce in vitro rooting of the Marubakaido rootstock and can replace the use of synthetic indoleacetic acid in the rooting of this cultivar.

## Introduction

Apples are a primary fruit species throughout the world, with an annual production of 64.3 million tons (Dobránszki & da Silva 2010). In 2009, Brazil produced 1.22 million tons over a cultivated area of approximately 39,000 hectares (IBGE, 2010). Due to the importance of this agribusiness, seedlings of high genetic and phytosanitary quality must be produced using efficient methods such as micropropagation (Abreu & Pedrotti, 2003).

Micropropagation has the primary advantage of producing disease-free plant clones and consists of a four-stage process: in vitro establishment, shoot multiplication, in vitro rooting of microcuttings, and acclimatization (George & Debergh, 2008).

*In vitro* rooting is a critical phase of micropropagation because depends on the genotype and physiological condition at the time of root induction (Martins & Pedrotti, 2001). The stage following rooting is called acclimatization and is necessary because the *in vitro* plants are cultivated under heterotrophic conditions (Hazarika, 2003). Morphological anomalies, such as non-functional stomata, and physiological anomalies, such as a decrease in photosynthesis, might occur during this process (Rogalski et al. 2003). These anomalies interfere with plant survival in the greenhouse or field. Therefore, acclimatization is critical for the transition of micropropagated plants from *in vitro* cultivation to *ex vitro* cultivation.

Most studies of apple *in vitro* rooting used synthetic auxins, such as indole butyric acid (IBA) and indoleacetic acid (IAA) with different rootstocks and scion cultivars. In contrast, acclimatization studies have focused on the use of different substrates, such as mixtures of peat and sand (3:1) for the M9 rootstock and peat, perlite and vermiculite (4:1:1) for the cultivars of Golden Delicious and Royal Gala (Dobránszki & da Silva 2010).

The *in vitro* rooting of plants using rhizobacteria has been studied previously in the micropropagation of olive and pine using *Pseudomonas* and *Agrobacterium rhizogenes*, respectively (Peyvandi et al. 2010; Villalobos-Amador et al. 2002). Acclimatization by the inoculation of arbuscular mycorrhizal fungi has also been successful (Locatelli & Lovato, 2002; Cavallazzi et al. 2007). The replacement of IAA, an easy-to-find and inexpensive auxin, with a rhizobia plant-growth promoting inoculant for the micropropagation of apple rootstocks, which is a time-consuming process, may provide a growth advantage under field conditions. However, this work did not study the development of plants under field conditions. The purpose of this study was to assess the use of inoculation with rhizobia of *Adesmia latifolia*, instead of synthetic IAA, to promote the rooting and acclimatization of Marubakaido apple (*Malus prunifolia* Borkh) micropropagated rootstock. Although this work does not evaluate the development of plants under field conditions, as far we know there are no reports in the literature evaluating the use of rhizobia for the rooting and acclimatization of apple *in vitro*. Therefore, this is the first study to evaluate plant-growth promoting rhizobia for rooting and acclimatization *in vitro* for the micropropagated rootstocks of apple trees.

## Materials and methods

Twenty rhizobia strains that were isolated from the root nodules of *Adesmia latifolia,* supplied by Epagri’s Laboratory of Biotechnology, were analyzed in terms of their production of the auxin IAA using a colorimetric method developed by (Asghar et al. 2002). The rhizobacteria isolates were grown on YMA (yeast-mannitol-agar) medium supplemented with tryptophan (50 mg L^-1^) for 72 hours at 28°C with agitation at 120 rpm. Next, a 50-μL aliquot of bacterial suspension was added to 96-well polystyrene microplates. A Salkovski reagent was then added, and the suspension was incubated at room temperature for 1 hour. A change in color from yellow to pink indicated the production of IAA in the reaction due to the oxidation of indole compounds by ferric salts. The concentration of IAA produced by bacteria was estimated by adjusting the regression curve obtained from the YMA incubation medium with known amounts of synthetic IAA (0, 25, 50, 100, and 150 μg L^-1^). The IAA concentration was determined using a wavelength of 492 nm on an ELx800 absorbance microplate reader (BioTek®). The experimental design was completely randomized with four replications. Rhizobia isolates with higher IAA production were used in the experiment for *in vitro* rooting and acclimatization of the Marubakaido apple rootstock. The research was conducted during October and November of 2010. Both the rooting and acclimatization experiments were carried out over 30 days.

The *in vitro* rooting experiment used explants of Marubakaido apple rootstock that were propagated according to the protocol described by (Giacobbo et al. 2003). Cultivation was conducted on MS medium (Murashige & Skoog 1962) with a reduction of 25% and 33% in ammonium nitrate and potassium nitrate, respectively. Therefore, the medium was composed of the following salts: potassium phosphate (0.17 g of KH_2_PO_4_. L^-1^), magnesium sulfate (0.37 g of MgSO_4_.7H_2_O. L^-1^), copper sulfate (0.025 mg of CuSO_4_.5H_2_O. L^-1^), iron sulfate (27.8 mg of FeSO_4_.7H_2_O. L^-1^), manganese sulfate (22.3 mg of MnSO_4_.4H_2_O. L^-1^), zinc sulfate (8.6 mg of ZnSO_4_.7 H_2_O.L^-1^), sodium molybdate (0.25 mg of Na_2_MoO_4_.2H2O. L^-1^), ammonium nitrate (1.24 g of NH_4_NO_3_. L^-1^), potassium nitrate (1.27 g of KNO_3_. L^-1^), calcium chloride (0.44 g of CaCl_2_.2H_2_O. L^-1^), sodium EDTA (37.3 mg of Na_2_EDTA. L^-1^), cobalt chloride (0.025 mg of CoCl_2_.6H_2_O. L^-1^) and potassium iodide (0.83 mg of KI.L^-1^). The carbon sources in this medium were sucrose (30 g. L^-1^) and myo-inositol (0.1 g. L^-1^), and vitamins were added, including nicotinic acid (0.5 mg. L^-1^), thiamin (0.1 mg. L^-1^), pyridoxine (0.5 mg. L^-1^), and glycine (2.0 mg. L^-1^). Agar (6 gL^-1^) was used as a gelling agent, and benzylaminopurine (0.8 mg BAP. L^-1^) was used as a growth regulator. The treatments consisted of explants inoculated with the following rhizobia: EEL0210, EEL16110, EEL16010B, EEL37810, and EEL16010B, which were grown in a liquid YMA medium with tryptophan. After determining the concentration of the IAA produced, an aliquot corresponding to a final concentration of 1 mg of IAA.L^-1^ was taken from the culture medium. This aliquot was added to the culture medium before and after autoclaving at 121°C for 15 minutes. Then, the broth was added to sterile culture medium at a temperature of 50°C to prevent the microorganisms from dying. Thus, a rooting culture media was used with dead (S) and living (L) rhizobia cells. Furthermore, two treatments without inoculation were used: one with the addition of 1 mg of synthetic IAA.L^-1^ and the other without IAA. The design was completely randomized with six replications of five plants.

The experiment used acclimatization explants that were rooted *in vitro* on an MS medium modified by (Giacobbo et al. 2003) without benzylaminopurine and with the addition of 1 mg of IAA.L^-1^. Similar to the previously mentioned experiment, the treatments were inoculated with a diluted suspension of rhizobia strains of EEL0210, EEL16110, EEL16010B, EEL37810, and EEL16010B to a concentration of 1 mg of IAA.L^-1^ in the substrate. In this experiment, we used a randomized block design with five replications of eight plants.

The variables analyzed in both experiments were shoot length (SL), root length (RL), number of primary roots (NPR), number of secondary roots (NSR), number of total roots (NTR), fresh-shoot biomass (FSB) and fresh-root biomass (FRB). The rooting rate was determined in the *in vitro* experiment, while the explant survival rate of the micropropagated Marubakaido apple rootstock was determined during the acclimatization experiment. The root length was measured using a method developed by (Tennant 1975) using a Petri dish divided into 1-cm squares. The roots were randomly arranged on a dish and the number of intersections within the lines of the squares were counted. The obtained value was incorporated into the following formula:

*RL* = *N* × *L* × 11/14, where RL = root length in cm; N = number of intersections; L = length of a side of the square.

The IAA production data in the *in vitro* rooting and acclimatization experiments were evaluated using analysis of variance, and the means were compared using the Scott-Knott test (p <0.05).

## Results

**Figure 1:**
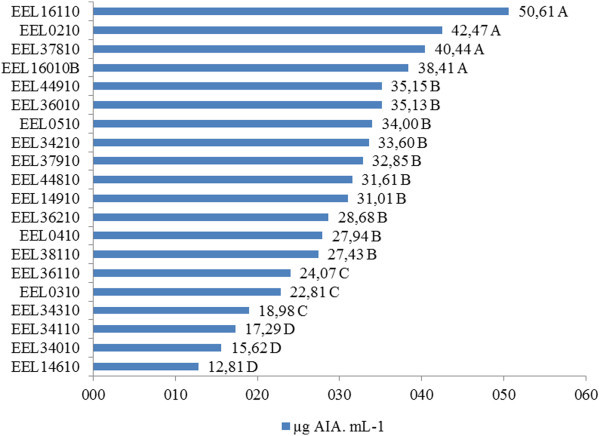
**The production of IAA by bacteria isolates from** ***A.*** ***latifolia*** **nodules (treatments with the same letter do not differ based on a Scott-Knott test (p <0.05).**

The rhizobia strains EEL16110, EEL0210, EEL37810, and EEL16010B showed higher *in vitro* IAA production, with values ranging from 38.4 to 50.6 mg IAA.mL^-1^. These were selected for the *in vitro* rooting and acclimatization experiments (Figure 1).

**Figure 2:**
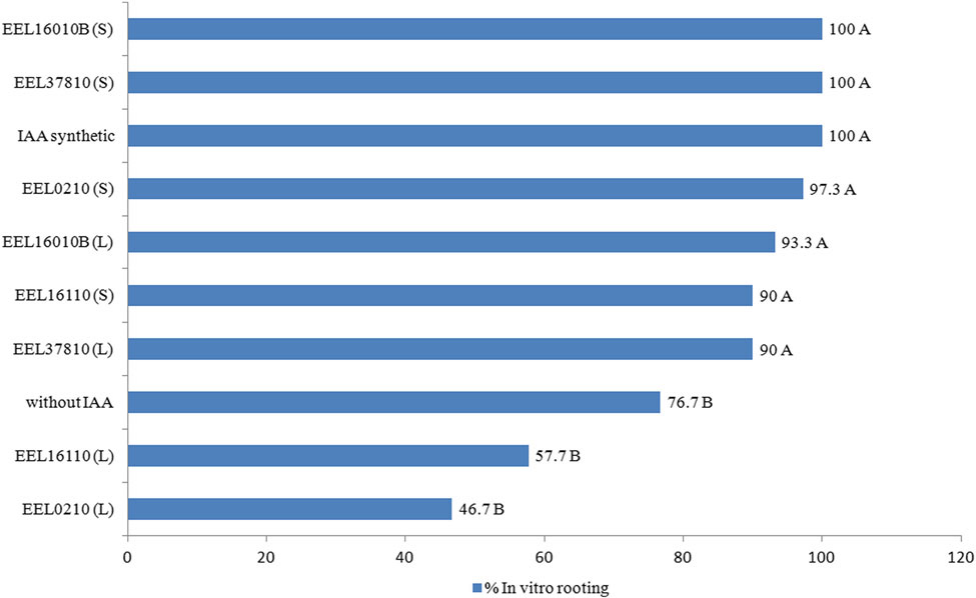
**The rooting rate of micropropagated Marubakaido apple rootstock using living (L) and dead (S) rhizobia of** ***A.*** ***latifolia*** **nodules (treatments with the same letter do not differ statistically based on a chi-squared test (p <0.05)).**

The explants were inoculated with the broth containing rhizobia isolates EEL37810 (S and L), EEL16010B (S and L), EEL0210 (S), and EEL16110 (S); those explants that received synthetic IAA showed higher *in vitro* rooting rates than the remaining treatments (Figure 2).

The explants inoculated with rhizobia and the ones treated *in vitro* with synthetic IAA did not show increases in the shoot length for the Marubakaido apple rootstock (Table 1).

**Table 1:** **Shoot length (SL), root length (RL), number of primary roots (NPR), number of secondary roots (NSR), number of total roots (NTR), fresh-shoot biomass (FSB), and fresh-root biomass (FRB) of micropropagated Marubakaido apple rootstock during** ***in vitro*** **rooting**

Treatment^*^	SL	RL	NPR	NSR	NTR	FSB	FRB
	(cm/plant)		(N°/plant)			(mg/plant)	
Control with synthetic IAA	2.4 ± 0.3 a	10.6 ± 3.7 b	5.3 ± 2.0 a	3.9 ± 1.7 a	9.2 ± 3.8 a	88.3 ± 17.5 a	31.4 ± 9.3 a
*Dead rhizobia*							
EEL0210 ^S^	2.4 ± 0.3 a	7.4 ± 2.6 c	4.4 ± 1.7 a	4.0 ± 1.7 a	8.4 ± 3.4 a	72.0 ± 14.3 b	19.1 ± 5.6 b
EEL16110 ^S^	2.5 ± 0.3 a	9.0 ± 3.1 b	3.6 ± 1.4 a	4.2 ± 1.8 a	7.7 ± 3.1 a	84.7 ± 16.8 a	21.8 ± 6.4 b
EEL16010B ^S^	2.9 ± 0.3 a	13.6 ± 4.7 a	5.4 ± 2.1 a	5.4 ± 2.4 a	9.8 ± 4.0 a	94.3 ± 18.7 a	32.7 ± 9.6 a
EEL37810 ^S^	2.5 ± 0.3 a	9.4 ± 3.3 b	4.3 ± 1.6 a	2.9 ± 1.3 a	7.1 ± 2.9 b	89.3 ± 17.7 a	18.1 ± 5.3 b
*Living rhizobia*							
EEL0210 ^L^	2.7 ± 0.3 a	3.3 ± 1.1 c	1.1 ± 0.4 b	2.7 ± 1.2 a	3.9 ± 1.6 b	58.2 ± 11.5 b	7.6 ± 2.2 c
EEL16110 ^L^	2.6 ± 0.3 a	5.7 ± 2.0 c	1.9 ± 0.7 b	3.2 ± 1.4 a	5.1 ± 2.1 b	64.1 ± 12.7 b	11.0 ± 3.2 c
EEL16010B ^L^	2.6 ± 0.3 a	13.1 ± 4.5 a	4.8 ± 1.8 a	6.1 ± 2.7 a	10.9 ± 4.5 a	100.6 ± 19.9 a	27.1 ± 8.0 a
EEL37810 ^L^	2.5 ± 0.3 a	10.3 ± 3.6 b	4.2 ± 1.6 a	4.8 ± 2.1 a	9.0 ± 3.7 a	91.9 ± 18.2 a	17.6 ± 5.2 b
Control without synthetic IAA	2.6 ± 0.3 a	6.7 ± 2.3 b	3.0 ± 1.1 b	3.6 ± 1.6 a	6.6 ± 2.7 b	85.7 ± 17.0 a	13.0 ± 3.8 c

*S= sterilized culture with dead cells; L = culture with live cells. Means with the same letter do not differ statistically based on a Scott-Knott test (p <0.05).

The rhizobia isolates EEL16010B (S and L), EEL37810 (S and L), EEL16110 (S), and EEL0210 (S) induced *in vitro* production of primary roots in the Marubakaido rootstock, similar to synthetic IAA. On the other hand, the induction of *in vitro* production of secondary roots was not significant. The rhizobia isolates EEL16010B (S and L), EEL37810 (L), EEL16110 (S), EEL0210 (S) and synthetic IAA induced the production of a greater number of roots *in vitro* than the other treatments.

The explants inoculated with broth containing cells of the isolate EEL16010B (S and L) produced longer roots than those inoculated with the other rhizobia studied (Table 1).

The rhizobia isolates EEL0210 (S and L) and EEL16110 (L) induced a lower production of fresh-shoot biomass, most likely because they produced lower root growth of micropropagated Marubakaido apple rootstock. The isolate EEL16010B (S and L) and the synthetic IAA induced higher production of fresh-root biomass than the other isolates (Table 1).

The studied rhizobia isolates did not promote the growth of apple explants in the acclimatization experiments (Table 2).

**Table 2:** **Shoot length (SL), root length (RL), number of primary roots (NPR), number of secondary roots (NSR), number of total roots (NTR), fresh-shoot biomass (FSB), and fresh-root biomass (FRB) of micropropagated Marubakaido apple rootstock during acclimatization**

Treatment*	SL	RL	NPR	NSR	NTR	FSB	FRB
	(cm/plant)		(N°/plant)			(mg/plant)	
EEL0210 ^S^	2.5 ± 0.2 a	10.6 ± 2.8 a	5.2 ± 0.6 a	8.0 ± 4.1 a	13.2 ± 4.7 a	145.5 ± 22.0 a	19.2 ± 2.8 a
EEL16110 ^S^	2.5 ± 0.2 a	11.3 ± 3.2 a	5.5 ± 1.6 a	8.9 ± 2.7 a	14.4 ± 4.3 a	165.4 ± 32.3 a	22.1 ± 6.6 a
EEL16010B ^S^	3.1 ± 0.4 a	14.1 ± 3.3 a	6.5 ± 1.8 a	10.5 ± 2.5 a	17.0 ± 4.2 a	175.3 ± 39.5 a	25.6 ± 7.7 a
EEL37810 ^S^	2.4 ± 0.4 a	12.7 ± 3.4 a	5.0 ± 1.1 a	10.1 ± 2.5 a	15.1 ± 3.6 a	140.2 ± 23.9 a	24.6 ± 8.4 a
Non-inoculated Control	2.6 ± 0.2 a	10.1 ± 3.2 a	5.3 ± 0.6 a	5. 8 ± 1.8 a	11.1 ± 2.4 a	143.2 ± 51.5 a	22.6 ± 10.0 a

* S= sterilized culture with dead cells; L = culture with live cells. Means with the same letter do not differ statistically based on a Scott-Knott test (p <0.05).

## Discussion

IAA production was observed in all rhizobium isolates studied, varying between 12.8 and 50.6 μg of IAA.mL^-1^. Phytohormone production, particularly of indoleacetic acid (IAA), by rhizobia has been identified as one of the mechanisms promoting growth in non-leguminous plants (Yanni et al. 2001; Chi et al. 2005) in terms of root growth, root hair proliferation, and the improvement of soil water and nutrient absorption by infected plants (Fuentes-Ramírez & Caballero-Mellado 2005). IAA production by rhizobia, ranging from 1.0 to 130.3 mg IAA.mL^-1^, was also observed in isolates of soybean rhizobia (Chen et al. 2002).

The rooting rates observed in this study in inoculated apple rootstock explants were similar to those reported by (Lima-Nishimura et al. 2003), who used different doses of indolebutyric acid (IBA).

The buds of the explants of the Marubakaido apple rootstock, including both the inoculated treatments and those receiving synthetic IAA *in vitro*, did not increase in length. This might have occurred because plant elongation is due to a synergistic action of gibberellins (Taiz & Zeiger, 2010). Although gibberellin production was not studied in this experiment, phytohormones other than IAA were also not studied; the lack of bud growth might indicate that the studied bacteria are not able to produce those phytohormones. The mechanism that regulates IAA production in rhizobacteria does not involve gibberellin production. The regulation of IAA production in rhizobacteria occurs through the signaling molecules of acyl-homoserine lactones (AHLs) that alter the gene expression of auxins in the host plant and increase its endogenous content (Mathesius et al. 2003). Therefore, there is a change in the hormone balance, which alters the development of the roots (Mathesius, 2010).

There was a higher number of primary roots in the explants of the Marubakaido apple rootstock inoculated *in vitro* with the broth of rhizobia isolates EEL16010B (S and L), EEL37810 (L), EEL16110 (S), and EEL0210 (S) and in those that received synthetic IAA. However, the same stimulatory phenomenon was not observed in the secondary roots. The number of primary roots observed in the present study was similar to the *in vitro* rooting of Marubakaido apple rootstock (Martins & Pedrotti, 2001) but was higher than that observed by (Lima-Nishimura et al. 2003) with different doses of IBA. However, the explants inoculated with living cells of rhizobia isolates EEL0210 (L) and EEL16110 (L) showed a smaller number of roots compared with the remaining treatments. In the case of the treatments inoculated with broths containing living cells, such differences in primary-root induction could be attributed to a greater or lesser degree of interaction between the living cells of the bacterial isolates with the micropropagated apple rootstock's genotype (Dodd et al. 2010). This interaction might lead to a decrease or increase in root growth (Remans et al. 2008). Inoculation of the broth (both sterile and containing living cells) of isolate EEL16010B in the explants resulted in the production of more elongated roots. The root-elongation values were superior to the ones obtained in the *in vitro* rooting experiments using synthetic auxins conducted by (Martins & Pedrotti 2001) and (Lima-Nishimura et al. 2003) for the Marubakaido apple rootstock, in which the root length per plant was 3.4 and 1.9 cm, respectively. A similar effect was obtained in the micropropagation of olive trees and of the medicinal plant *Chlorophytum borivilianum* inoculated with *Pseudomonas* sp., *Pseudomonas fluorescens* and *Piriformospora indica* (Peyvandi et al. 2010; Gosal et al. 2010).

The lower production of fresh-shoot biomass in the explants of micropropagated Marubakaido apple rootstock inoculated with the broth of rhizobia isolates EEL0210 (S and L) and EEL16110 (L) most likely resulted from the smaller root growth observed in these treatments. The isolate EEL16010B (S and L) and synthetic IAA induced a higher production of fresh-root biomass than the other isolates (Table 1). This positive effect on the production of fresh-root biomass was also observed in the micropropagation of *Phoitinia* inoculated with different species of *Azospirillum* (Larraburu et al. 2007).

Root-growth stimulation by the microbial isolates was most likely due to an interaction between the genotype of the plant and of the microorganism. This interaction regulates the plant’s response to the auxin (IAA) and its production by the microorganism. Thus, growth was induced by alterations in the root elongation and architecture of the micropropagated Marubakaido apple rootstock (Dodd et al. 2010).

No differences were observed between the treatments in the acclimatization experiment, most likely due to the short period of observation. Other studies in the literature were conducted over up to 90 days (Thomas et al. 2010). Despite the fact that this experiment did not observe variations between the inoculated treatments and the non-inoculated control, it is not possible to discard the hypothesis that inoculation with rhizobia can be efficient. In other studies, the positive effect of inoculation with plant-growth promoting bacteria has already been established. The acclimatization experiment of *Camellia sinensis* inoculated with *Azospirillum* showed changes in the shoot length and fresh weight after a period of 90 days (Thomas et al. 2010). (Oliveira et al. 2010) observed an increase in the shoot and fresh-root weight of *Zingiber spectabile* inoculated with *Bacillus pumillus* after three months. In trials with rootstocks of *Prunus,* plant height effects were observed after a period of between 90 and 150 days after inoculation (Bonaterra et al. 2003).

Our results showed that phyto-stimulating substances were present and active in the EEL1610B broth, even after sterilization by autoclaving. This shows that these substances withstood the heat treatment and that they can become a source of growth factors that may be used for rooting of micropropagated Marubakaido apple rootstock.

The rhizobia isolate EEL1610B can be used to induce *in vitro* rooting of the Marubakaido apple rootstock. In addition, the broth containing IAA produced by the EEL1610B can be used instead of synthetic indoleacetic acid for the *in vitro* rooting of the Marubakaido apple rootstock.
